# Paradoxical Immune Phenotypes and Dual-State Immune Regulators in Plants: The GSL5 Case Study

**DOI:** 10.3390/ijms27125375

**Published:** 2026-06-15

**Authors:** Lixia Gao, Rong Zuo, Xiong Zhang

**Affiliations:** Key Laboratory of Biology and Genetic Improvement of Oil Crops, Ministry of Agriculture and Rural Affairs, Oil Crops Research Institute, Chinese Academy of Agricultural Sciences, Wuhan 430062, China; 15172315586@163.com (L.G.); hu086zr@163.com (R.Z.)

**Keywords:** plant immunity, paradoxical resistance, dual-state immune regulators, immune homeostasis, resistance breeding

## Abstract

Plant immune genes are traditionally classified as resistance genes, susceptibility genes, or positive/negative regulators of defense. However, this framework does not fully explain a subset of immune-associated genes that display paradoxical disease phenotypes, in which genetic disruption enhances resistance despite the normal involvement of these genes in defense-related processes. *GSL5/PMR4* is a representative example. As a pathogen-induced callose synthase, *GSL5* contributes to papillary callose deposition and structural defense. Paradoxically, loss of *GSL5* confers resistance to powdery mildew through salicylic acid- and N-hydroxypipecolic acid-associated pathways, as well as broad-spectrum resistance to *Plasmodiophora brassicae* through jasmonic acid-dependent immunity. Here, we refer to such genes as dual-state immune regulators, whose functional presence and genetic disruption promote resistance through distinct immune states. Similar regulatory patterns have been reported in several immune-related processes, including MAPK signaling, calcium influx, membrane trafficking, and receptor-proximal immune signaling. Representative examples include the MEKK1–MKK1/MKK2–MPK4 module, CNGC2/CNGC4, EXO70B1 and BIK1. This review uses GSL5 as a central example to discuss paradoxical immune phenotypes and dual-state immune regulators in plants, focusing on their biological features, potential mechanisms, and implications for resistance breeding.

## 1. Introduction

Plants encounter diverse microbial pathogens throughout their life cycle and rely on a multilayered immune system to detect invasion, restrict colonization and maintain cellular integrity [1,2,3]. Surface-localized pattern-recognition receptors perceive conserved microbe- or pathogen-associated molecular patterns (MAMPs/PAMPs), as well as damage-associated molecular patterns (DAMPs). This perception activates pattern-triggered immunity (PTI), whereas intracellular immune receptors detect pathogen effectors or their activities and induce effector-triggered immunity (ETI) [4,5]. These immune layers are supported by cell wall reinforcement, calcium signaling, mitogen-activated protein kinase (MAPK) cascades, hormone signaling pathways, membrane trafficking, transcriptional reprogramming and antimicrobial outputs [1,2,3,4,5]. Within this framework, immune-associated genes are often described as positive regulators that promote resistance, negative regulators that restrain defense, resistance genes that recognize pathogens, or susceptibility genes that are required for pathogen compatibility [4,5,6,7].

This classification has been valuable, especially for breeding and genetic studies. Classical susceptibility genes provide a clear example, where their loss can block pathogen colonization because a host factor required by the pathogen is removed [6,7]. Mildew resistance locus O (*MLO*) genes are representative susceptibility genes whose loss confers powdery mildew resistance [8]. Sugars Will Eventually be Exported Transporter (*SWEET*) genes provide another example, as blocking their TAL effector-induced expression can confer durable disease resistance in crops [9]. However, some resistant phenotypes after disruption of immune-associated genes do not fit this straightforward model. In some cases, the disrupted gene is not an obvious pathogen-required factor but an immune-associated regulator with recognizable roles in defense, signaling or cellular homeostasis.

*GSL5/PMR4* represents a particularly informative example. *GSL5* encodes a callose synthase responsible for pathogen- and wound-induced callose deposition, a classical defense-associated cell wall response [10,11]. Notably, *pmr4*/*gsl5* mutants exhibit resistance to powdery mildew despite their impaired pathogen-induced callose deposition. This resistance largely depends on salicylic acid (SA)-associated immunity [10]. Subsequent studies showed that GSL5 inactivation confers broad-spectrum resistance to *Plasmodiophora brassicae* pathotypes in cruciferous plants through a distinct immune route involving jasmonic acid (JA)-associated resistance during secondary infection [12]. Thus, GSL5 cannot be simply viewed either as a positive defense factor or a susceptibility gene. The resulting disease outcome depends on pathogen context, immune pathway balance and whether the host process is used protectively or exploited by the pathogen. We refer to such genes as dual-state immune regulators because their functional presence and genetic disruption can promote distinct immune states.

Similar complexity has been observed in other immune-associated regulators, including the MEKK1 (MAPKKK1)–MKK1/MKK2 (MAPKK1/2)–MPK4 (MAPK4) module [13,14], DND1/CNGC2 (Defense, No Death 1/Cyclic Nucleotide-Gated Channel 2) and DND2/CNGC4 (Defense, No Death 2/Cyclic Nucleotide-Gated Channel 4) [15], EXO70B1 (Exocyst complex component of 70 kDa B1) [16] and BIK1 (*Botrytis*-induced kinase 1) [17]. These genes participate in early immune signaling, calcium influx, membrane trafficking or defense regulation. Yet their disruption can sometimes lead to retained or enhanced resistance through compensatory immunity, immune surveillance, or altered pathway balance [13,14,15,16,17]. In this review, we use *GSL5* as a central example to discuss this class of dual-state immune regulators. We summarize how their normal functions, mutant phenotypes and pathogen interactions challenge simple gene classifications. Finally, we consider how this view may inform future studies of plant immunity and resistance breeding.

## 2. Dual Roles of GSL5 in Plant Immunity

### 2.1. GSL5 as an Inducible Callose Synthase with Broader Biological Functions

Callose deposition represents one of the best-characterized inducible responses during plant–pathogen interactions. It accumulates at attempted penetration sites and contributes to papillae and cell wall appositions, where callose acts in concert with antimicrobial compounds, reactive oxygen species, phenolics and defense-related proteins to reinforce local defense interfaces [11,18,19]. According to this classical view, pathogen-induced callose is considered as a structural barrier that restricts microbial entry.

In *Arabidopsis*, GSL5/PMR4/CALS12 represents the major callose synthase responsible for wound- and pathogen-induced callose deposition [10,20,21]. Among the 12 glucan synthase-like (GSL) proteins encoded in the *Arabidopsis* genome, GSL5 is closely associated with papillary callose formation during pathogen challenge [10,21]. Genetic analyses have shown that *pmr4*/*gsl5* mutants are strongly impaired in wound- and pathogen-induced callose accumulation, establishing GSL5 as a central enzyme for inducible callose synthesis [10,20]. Consistent with this role, enhanced early callose deposition can increase penetration resistance against powdery mildew fungi [22].

GSL5-associated callose deposition is not limited to pathogen challenge. Recent work has shown that PMR4 and plasmodesmata-located proteins contribute to β-aminobutyric acid (BABA)-primed penetration resistance against *Hyaloperonospora arabidopsidis*. This indicates that PMR4-dependent callose participates in chemically primed cell wall defense [23]. PMR4 has also been linked to abiotic adaptation. Under phosphate deficiency, PMR4-dependent callose deposition is induced in root hairs. This deposition contributes to phosphate acquisition and shoot growth, independently of defense-associated SA accumulation [24]. These observations broaden the biological scope of GSL5. GSL5 therefore functions as a pathogen-responsive callose synthase and as a regulator of inducible cell wall status, immune priming and stress adaptation.

From a classical physiological perspective, disruption of GSL5 would be expected to weaken structural defense or stress adaptation. Unexpectedly, loss of GSL5 can instead produce strong resistance in certain disease contexts.

### 2.2. Loss of GSL5 Activates Resistant Immune States

The classical paradigm of GSL5 was challenged by the discovery that *pmr4*/*gsl5* mutants are resistant to powdery mildew despite their strong defect in pathogen-induced callose deposition [10]. This resistance cannot be attributed to structural reinforcement. Instead, it depends largely on SA-associated immunity, as blocking SA accumulation or SA signaling restores susceptibility in *pmr4*/*gsl5* mutants [10]. Subsequent studies revealed that loss of GSL5 leads to constitutive defense activation, elevated defense marker expression and altered defense metabolism [25,26]. More recently, N-hydroxypipecolic acid (NHP)-associated amplification was shown to contribute to the autoimmune-like state caused by loss of PMR4/GSL5 [26].

GSL5 inactivation also confers resistance in a distinct pathosystem. In cruciferous plants, loss of GSL5 provides broad-spectrum resistance to diverse *P. brassicae* pathotypes [12]. Consistent with this model, an independent study recently reported that loss of PMR4 callose synthase confers JA-dependent resistance to clubroot disease in both *Arabidopsis* and *Brassica napus* [27], further supporting PMR4/GSL5 as a key example of pathogen-dependent immune rewiring and as a potential gene-editing target for clubroot resistance improvement in Brassicaceae crops. Unlike powdery mildew resistance, which is mainly associated with SA/NHP-related immunity, clubroot resistance in the *gsl5* mutant depends largely on JA-associated immunity during secondary infection [12]. This distinction is important because it shows that GSL5 loss does not activate a single fixed defense route. Instead, the resulting resistant state depends on the pathogen lifestyle, infected tissue and infection stage.

### 2.3. GSL5 as a Representative Dual-State Immune Regulator

The clubroot pathosystem further deepens the GSL5 paradox. GSL5 acts as a defense-associated callose synthase whose loss triggers compensatory immunity, and it can also help establish a pathogen-compatible state during *P. brassicae* infection [12]. The underlying mechanism is discussed in detail in Section 4.3. At this stage, it is worth noting that this single host protein can contribute to inducible callose deposition in one context, support stress-related cell wall adaptation in another, and become linked to disease compatibility in a specific pathogen system.

Therefore, the GSL5 paradox is not simply that callose loss enhances resistance. More broadly, GSL5 demonstrates how an immune-associated host process can shift among structural defense, immune priming, stress adaptation and pathogen-dependent compatibility states. This makes GSL5 a useful entry point for discussing dual-state immune regulators, whose normal functions are biologically important but whose disruption can redirect the plant into a distinct resistant state.

## 3. Beyond GSL5: Paradoxical Resistance Phenotypes in Plants

GSL5 is not unique in this regard. Several immune-associated regulators also exhibit disease phenotypes that cannot be readily explained by a simple positive- or negative-regulator model. These genes are not identical to *GSL5*, but they similarly illustrate how disruption of a defense-related process may retain or even enhance resistance in particular contexts.

### 3.1. MPK4 and the MEKK1–MKK1/MKK2–MPK4 Module

MPK4 belongs to the conserved MAP kinase family and contains a typical Ser/Thr kinase domain with a TEY activation motif [28]. Interest in MPK4 initially arose from *Arabidopsis mpk4* mutants, which show constitutive systemic acquired resistance, elevated SA levels, constitutive expression of pathogenesis-related genes, and enhanced resistance to virulent pathogens [13]. Subsequent studies placed MPK4 in the MEKK1–MKK1/MKK2–MPK4 cascade, a MAPK module activated during early immune signaling [29,30]. Similar phenotypes were also observed in *mekk1* and *mkk1 mkk2* mutants, including spontaneous cell death and increased defense gene expression [29,30]. Therefore, this module is associated with immune signaling in its normal state, but its disruption can also trigger strong defense activation.

### 3.2. DND1/CNGC2 and DND2/CNGC4

DND1/CNGC2 and DND2/CNGC4 are plasma membrane-localized cyclic nucleotide-gated channels that are proposed to assemble into a heteromeric channel complex [31,32,33]. Both proteins contain typical CNGC features, including multiple transmembrane regions and C-terminal cyclic nucleotide-binding and calmodulin-binding domains [31,33]. In *Arabidopsis*, the CNGC2–CNGC4 channel has been linked to early immune-associated Ca^2+^ signaling and pathogen defense [31,32,33]. Nevertheless, the corresponding *dnd1*/*cngc2* and *dnd2*/*cngc4* mutants show reduced hypersensitive response (HR)-associated cell death after avirulent pathogen recognition, while their disease resistance is retained or enhanced [32,34]. These mutants therefore uncouple impairment of a specific immune output from the overall resistant state.

### 3.3. EXO70B1

EXO70B1 is a member of the plant-expanded EXO70 family [35,36]. In *Arabidopsis*, EXO70B1 is closely related to EXO70B2, and both proteins are implicated in immune-related trafficking and PTI responses induced by MAMPs [37,38]. Consistent with this role, young *exo70B1* mutant plants show reduced early PTI responses and enhanced susceptibility to *Pseudomonas syringae* pv. tomato DC3000 [37,38]. However, the phenotype of *exo70B1* mutants is not limited to immune deficiency. Loss of EXO70B1 also causes ectopic HR-like cell death, altered autophagy-related trafficking, and enhanced resistance to a range of pathogens [16,36,39]. EXO70B1 therefore links immune trafficking with both defense competence and abnormal immune activation.

### 3.4. BIK1

BIK1 is a plasma membrane-associated receptor-like cytoplasmic kinase (RLCK) belonging to the RLCK VII subfamily [40,41]. As a receptor-proximal kinase, it functions in early PTI signaling and also connects with brassinosteroid-related growth signaling [40,41,42]. Consistent with its immune role, *bik1* mutants are more susceptible to necrotrophic fungi such as *Botrytis cinerea* and *Alternaria brassicicola* [17]. Conversely, *bik1* mutants have also been reported to show enhanced resistance under certain conditions [17]. BIK1 therefore reveals how the disease outcome of disrupting an immune-associated kinase can depend on pathogen lifestyle and signaling context.

**Figure 1:**
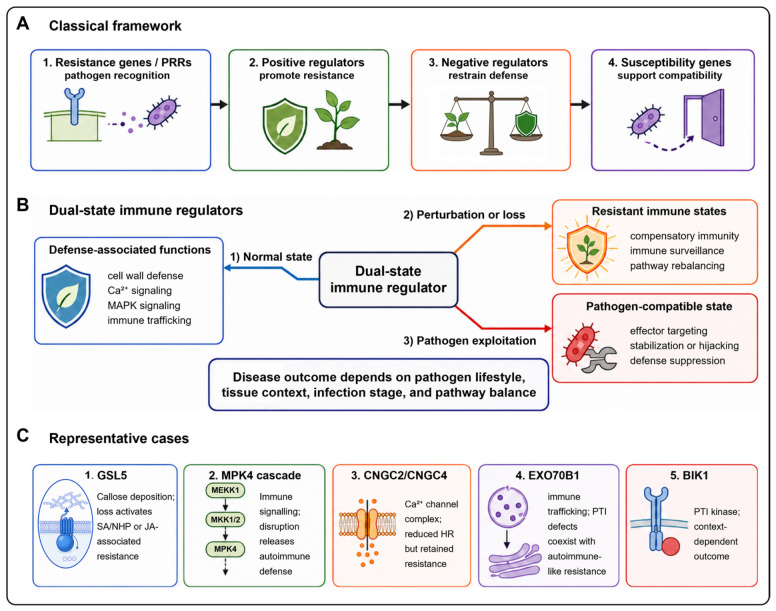
Conceptual model of dual-state immune regulators. (**A**) According to the conventional view, immune-related genes are often grouped as positive regulators, negative regulators or susceptibility factors according to their effects on disease resistance. (**B**) A subset of genes does not conform to this positive/negative/susceptibility-factor classification. They have normal functions in defense-related processes, yet their disruption or pathogen-mediated perturbation can shift the plant into a different immune state, thereby conferring resistance rather than susceptibility. (**C**) GSL5/PMR4, the MPK4 module, CNGC2/CNGC4, EXO70B1 and BIK1 represent different forms of this dual-state behavior. Their phenotypes depend on pathogen lifestyle, tissue context, infection stage and pathway balance.

Together, these cases suggest that some immune-associated genes cannot be fully interpreted by a linear positive- or negative-regulator model. However, these mutants differ markedly in the severity of autoimmune phenotypes and associated fitness costs. For example, *mpk4* mutants show strong constitutive defense activation accompanied by extreme dwarfism [13,29,30], whereas *dnd1*/*dnd2* mutants generally display more moderate growth and fitness penalties [15,32,34]. In comparison, *exo70b1* mutants exhibit relatively milder senescence-associated phenotypes [16,36,39]. Thus, dual-state immune regulators should not be viewed as a uniform class with equivalent agronomic risks. Instead, their disease phenotypes depend on the immune state established by normal function, genetic disruption or pathogen-mediated perturbation (Figure 1).

## 4. Mechanistic Basis of Paradoxical Resistance

The examples discussed above raise a common question of why disruption of an immune-associated gene can confer resistance instead of susceptibility. The answer is unlikely to involve a single mechanism. In most cases, the mutant phenotype reflects a shift in immune state, rather than the simple disappearance of one defense component. Several non-mutually exclusive mechanisms appear particularly relevant (Figure 1).

### 4.1. Compensatory Immune Activation

A recurring feature of paradoxical resistance mutants is the activation of compensatory immune pathways. In *pmr4*/*gsl5*, loss of pathogen- and wound-induced callose deposition is accompanied by enhanced resistance to powdery mildew through SA/NHP-associated immunity [10,26]. In clubroot disease, the same gene loss leads to a different resistance state, mainly dependent on JA-associated immunity during *P. brassicae* infection [12]. A related separation between immune outputs is seen in *dnd1*/*cngc2* and *dnd2*/*cngc4*. These mutants were originally identified by their reduced hypersensitive cell death following recognition of avirulent *P. syringae*, whereas they retain or show enhanced disease resistance [32,34]. CNGC2 and CNGC4 form a heteromeric channel involved in early PAMP- and DAMP-associated Ca^2+^ signaling, including flg22-, Pep3- and extracellular ATP (eATP)-related immune responses [33,43]. However, loss of these channels is accompanied by constitutive SA-associated defense, elevated expression of pathogenesis-related (PR) genes, and enhanced resistance to multiple pathogens [15,32,34]. Expression of *NahG* suppresses the enhanced resistance and PR gene expression but does not restore the HR defect, indicating that cell death and SA-dependent resistance are genetically separable in these mutants [15]. These cases indicate that the immune outputs impaired in the mutants are distinct from the immune states responsible for resistance.

### 4.2. Immune Buffering and Defense Restraint

The MEKK1–MKK1/MKK2–MPK4 module reveals how an immune pathway can act in two directions. This MAPK cascade is activated during MAMP-triggered immunity and contributes to basal defense and MAMP-induced gene expression [13,29,44]. At the same time, it suppresses inappropriate immune activation. Disruption of *MEKK1*, *MKK1*/*MKK2* or *MPK4* in plants leads to spontaneous cell death, constitutive defense gene expression and autoimmune-like phenotypes [13,29,30,44]. This phenotype is not simply a consequence of impaired PTI signaling. The leucine-rich repeat receptor (NLR) protein SUMM2 monitors the integrity of the MPK4 cascade through CRCK3, whose phosphorylation is reduced when the cascade is disrupted [14,29]. Subsequent studies showed that disruption of this cascade activates the HOP1–LET7 module, which promotes stabilization of SUMM2 and triggers SUMM2-mediated autoimmunity [45]. EXO70B1 exhibits a similar buffering mechanism in membrane trafficking. Under normal conditions, EXO70B1 contributes to immune-related trafficking and FLS2 homeostasis at the plasma membrane [36,38]. However, loss of EXO70B1 activates HR-like cell death and enhanced resistance through TN2- and CPK5-associated defense activation [16,39]. This suggests that immune buffering is not limited to classical signaling enzymes. Cellular systems that support regulated defense can also be monitored, including MAPK signaling and membrane trafficking. They may shift the plant from controlled immune responsiveness to constitutive defense activation once disturbed.

### 4.3. Pathogen Exploitation of Immune-Associated Processes

Pathogens do not simply encounter immune-associated host processes as fixed defense modules. During infection, effectors can stabilize, degrade, mislocalize or redirect these processes, thereby establishing a pathogen-compatible state. The GSL5–clubroot pathosystem provides a direct example. During *P. brassicae* secondary infection, the effector PbPDIa interacts with and stabilizes GSL5, thereby maintaining a host state favorable for cortical infection and suppression of JA-associated resistance [12]. Thus, inactivation of GSL5 eliminates its callose-synthase function and removes a pathogen-exploited host component. Other immune-associated host factors can also become targets of pathogen manipulation. EXO70B1 contributes to PTI-related trafficking, but AvrPtoB promotes its ubiquitination and degradation, while RipE1 targets EXO70B1/RIN4 in a process monitored by Ptr1 [38,46]. BIK1-related RLCKs are also recurrent effector targets: fungal NIS1 inhibits BAK1/BIK1 kinase functions, XopR associates with BIK1 and other RLCKs, Avr2 interferes with BIK1 mono-ubiquitination and plasma membrane dissociation, and RipAV promotes BIK1 degradation through the CPK28–PUB module [47,48,49,50]. Although these mechanisms differ, they converge on a common point, namely that immune-associated host processes can serve as compatibility factors, effector targets, or guarded components.

### 4.4. Pathogen Context and Pathway Balance

The outcome of disrupting an immune-associated regulator is strongly shaped by pathogen lifestyle and pathway balance. GSL5 provides an illustrative example, as its loss confers resistance to powdery mildew via the SA/NHP pathway but resistance to clubroot disease via the JA pathway [10,12,26]. Thus, GSL5 loss does not activate a single fixed defense route; instead, it shifts immune balance in a pathogen-dependent manner. BIK1 provides another example of this context dependence. BIK1, as an early PTI-associated kinase, contributes to resistance against necrotrophic fungi such as *B. cinerea* and *A. brassicicola*, yet enhanced resistance to virulent *P. syringae* has been reported in *bik1* under certain conditions [17]. This difference is consistent with the broader role of BIK1 at the crossroads of immune signaling and brassinosteroid-related growth regulation [42]. Pathogens can further reshape this balance. For example, *P. syringae* AvrB activates MPK4- and RIN4-dependent JA responses to enhance susceptibility, illustrating how a defense-related hormone pathway can become favorable to a particular pathogen when placed in the wrong signaling context [41]. These observations suggest that paradoxical resistance is not an intrinsic property of a gene, but a phenotype emerging from the interaction between gene function, pathogen lifestyle, hormone balance, and tissue context.

## 5. Reconsidering Immune Gene Classification in Plants

Plant immune genes are commonly assigned relatively clear functional labels. Resistance genes and pattern-recognition receptors detect pathogen molecules and activate defense; whereas positive regulators transmit or amplify immune signals, negative regulators restrain excessive defense, and susceptibility genes provide host functions required for pathogen colonization [4,5,7]. These categories remain valuable, especially when describing classical recognition events or identifying breeding targets. For example, loss of a true susceptibility gene usually reduces disease because a pathogen-required host factor is removed [7]. In these cases, the biological interpretation is relatively direct.

Unlike classical susceptibility genes, dual-state immune regulators are not defined solely by their requirement for pathogen compatibility but by the coexistence of host-beneficial functions and mutation-induced resistant states. GSL5 contributes to pathogen-induced callose deposition [10,25], MPK4 participates in MAMP-triggered signaling [29,30], CNGC2/CNGC4 mediate early Ca^2+^ responses [33], EXO70B1 supports immune-related trafficking [36,38], and BIK1 acts as a central RLCK in PTI [40,42]. These are immune-associated functions in the normal plant. Their resistant loss-of-function phenotypes, therefore, cannot be interpreted simply as removal of a susceptibility factor. In many cases, resistance may arise when gene disruption changes the immune state, allowing SA/NHP or JA pathways to become dominant, NLR-mediated surveillance to be released, membrane trafficking to be perturbed, or a pathogen-exploited host process to be eliminated [10,12,14,26,38,45].

This distinction is important because the same resistant phenotype may arise from different biological explanations. A resistant mutant may reflect compensatory immunity, as in *pmr4*/*gsl5* powdery mildew resistance; pathogen exploitation, as illustrated by the PbPDIa–GSL5 interaction during clubroot secondary infection; or immune surveillance, as found in the MPK4–SUMM2 and EXO70B1–TN2 systems [10,12,14,26,45]. These mechanisms should not be merged into one broad category of loss-of-function resistance. For this type of gene, a more productive question is what immune state is generated when the gene is functional, disrupted, or manipulated by a pathogen, rather than whether it acts as a positive or negative regulator.

This perspective also changes how we approach such genes. Endpoint disease scoring alone is not sufficient. A resistant mutant at the final disease stage could have lost an early defense output, activated a late compensatory pathway, blocked a pathogen-specific compatibility step, or developed an autoimmune-like state. Time-course infection assays, pathogen biomass measurements, tissue-specific observations, hormone profiling, defense marker expression, cell wall readouts and cell death assays are needed to separate these possibilities. For GSL5-like regulators, genetic epistasis is especially important. Crossing into SA-, JA-, ethylene (ET)-, NHP-, PAD4/EDS1- or NLR-defective backgrounds can reveal whether resistance depends on compensatory signaling, immune surveillance or loss of pathogen compatibility.

Mechanistic assays are equally necessary. Transcriptomic or metabolomic changes may indicate immune reprogramming, yet they cannot explain why resistance occurs. Protein interaction assays, effector–target validation, protein stability measurements, phosphorylation or ubiquitination assays, and complementation with domain or activity mutants are often needed. This is well illustrated by recent examples, in which HopAI1 targeting of MPK4 activates SUMM2-mediated immunity, HOP1–LET7–CRCK3 provides a molecular link between MPK4 cascade disruption and SUMM2 activation, AvrPtoB and RipE1 target EXO70B1/RIN4-associated trafficking nodes, and several bacterial or fungal effectors target BIK1-related PTI signaling by altering phosphorylation, ubiquitination, localization or protein stability [14,38,45,46,47,49,50]. Such details are critical because they distinguish a defense regulator from a guarded node or a pathogen-exploited factor.

A practical classification for these genes may therefore need to be state-based rather than label-based. Under normal conditions, a gene typically supports defense or immune homeostasis. During infection, a pathogen may exploit the same process. After mutation, the plant may enter a different immune state that restricts a particular pathogen. This perspective also explains why broad resistance and fitness costs often co-occur. Specifically, strong SA activation, NLR activation, altered trafficking or disrupted hormone balance may restrict disease but also affect growth, development or resistance to other pathogens [51]. For crop improvement, the central question is therefore whether the resistant state is stable, pathogen-relevant and separable from unacceptable developmental or susceptibility costs, rather than simply whether gene disruption increases resistance.

## 6. Implications for Crop Resistance Improvement

Dual-state immune regulators present both opportunities and risks for crop resistance improvement. As this review shows, not all resistant mutants are equally suitable for breeding, especially when the affected genes also retain useful functions in the normal plant. Among the dual-state regulators discussed here, GSL5 currently provides the strongest application case. Its inactivation confers broad-spectrum resistance to multiple *P. brassicae* pathotypes in cruciferous plants, and the resistance is linked to a defined infection stage and a specific immune pathway [12]. By contrast, MPK4, CNGC2/CNGC4, EXO70B1 and BIK1 are better viewed at present as mechanistic examples rather than direct knockout targets, because they are deeply connected with basal defense, immune surveillance, calcium signaling, membrane trafficking, cell death, growth regulation or responses to other pathogens [13,14,15,16,17].

In breeding, the edited allele is as important as the target gene. In rice, different edits of the susceptibility gene *RBL1* produce sharply different impacts. For example, a loss-of-function allele confers broad resistance but causes severe yield loss, whereas a CRISPR-generated 12 bp deletion provides resistance to multiple pathogens without an obvious yield penalty [52]. A similar trade-off applies to *ROD1*, where complete disruption enhances disease resistance but affects growth, whereas a natural point variant improves resistance with limited agronomic cost [53]. Collectively, these cases suggest that gene disruption to achieve resistance is not a single strategy. For genes with critical host functions, precise alleles are generally preferable to null alleles.

**Figure 2:**
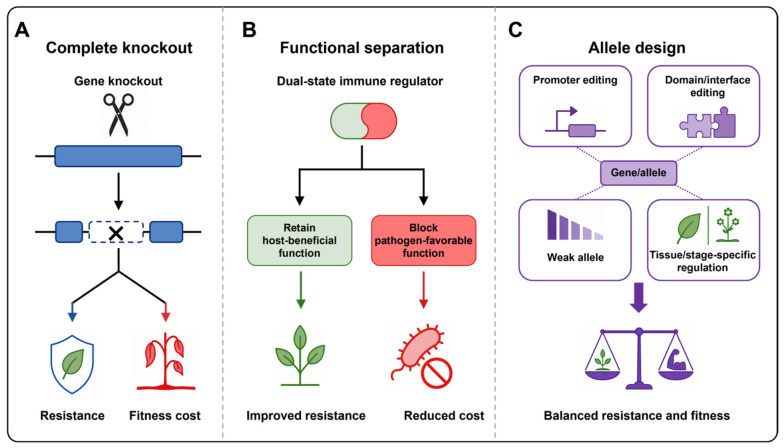
Application strategies for dual-state immune regulators. (**A**) Complete knockout can remove a pathogen-favorable host factor and generate disease resistance, but may also eliminate useful host functions and cause fitness or developmental costs. (**B**) Functional separation aims to retain the host-beneficial activity of a dual-state immune regulator while blocking the branch that is exploited by pathogens or linked to susceptibility. This strategy may improve resistance with lower fitness cost. (**C**) Allele design provides more refined options, including promoter editing, domain or interaction-interface editing, weak alleles, and tissue- or stage-specific regulation. These approaches may help balance disease resistance with normal growth and agronomic performance.

For dual-state regulators, the long-term goal should be functional separation. GSL5 makes this point clearly. It plays a role in defense-associated callose deposition under certain conditions, whereas during clubroot infection it can be part of a pathogen-favorable state. The ideal allele would retain useful cell wall or stress-related functions, while severing the pathogen-exploited branch. In the GSL5–clubroot system, this could involve editing the PbPDIa-interacting region, modifying infection-stage or tissue-specific regulation, or altering downstream connections that suppress JA-associated resistance, rather than abolishing GSL5 activity in all tissues and contexts [12]. This is conceptually similar to efforts to reduce growth–defense trade-offs. The goal is not to maximize immune activation but rather to identify points where resistance can be uncoupled from developmental cost [51,54]. These application strategies are summarized in Figure 2.

Currently, several successful breeding strategies provide useful precedents. Editing effector-binding elements in rice SWEET promoters prevents TAL effector-induced expression during bacterial blight infection while preserving the normal coding function of sugar transporter genes [9,55]. Pathogen-inducible expression is another strategy. Pathogen-responsive upstream open reading frames (uORFs) from *TBF1* enable translational control. In *Arabidopsis* and rice, this system drives NPR1 protein accumulation mainly during infection, thereby improving resistance without obvious yield penalties [56]. In addition, the rice *Pigm* locus contains the paired genes *PigmR* and *PigmS*. *PigmR* confers broad-spectrum blast resistance but can reduce yield, whereas *PigmS* offsets that cost in a tissue-specific manner, allowing strong leaf resistance while maintaining grain yield [57]. These examples show that useful resistance often depends more on when, where and how strongly a gene is active than on whether it is present. Beneficial microbes may also complement genetic strategies by shaping plant-associated microbial communities and supporting crop performance under field-relevant conditions [58,59].

Combinatorial editing may also be useful, but it should be applied with caution. Simultaneous editing of rice susceptibility genes such as *Pi21*, *Bsr-d1* and *Xa5* or combinations involving *Bsr-d1*, *Pi21* and *ERF922* can broaden resistance to different pathogen groups [60,61]. However, some immune-associated genes have opposite effects in different pathosystems. For instance, OsBZR1 positively regulates resistance to the necrotrophic pathogen *Rhizoctonia solani* but negatively regulates resistance to the hemibiotrophic pathogen *Xanthomonas oryzae* pv. *oryzae* (Xoo) [62,63], whereas OsMKP1 positively regulates vascular defense against Xoo but negatively regulates resistance to the mesophyll pathogen *Xanthomonas oryzae* pv. *oryzicola* (Xoc) [64]. These examples highlight the need to test resistance alleles against pathogen diversity, rather than against a single isolate or disease.

For dual-state immune regulators, practical deployment should therefore follow a stepwise route. Initially, it is necessary to determine whether resistance arises from loss of pathogen compatibility, compensatory immunity, immune surveillance, or pathway rebalancing. Subsequently, it should be tested whether the resistant phenotype can be reproduced by partial editing, promoter editing, domain editing or inducible regulation. Finally, candidate alleles should be evaluated using pathogen diversity panels, time-course infection assays, yield-related traits and multi-environment trials. At present, GSL5 represents the most promising breeding-related target among the genes discussed here, whereas MPK4, CNGC2/CNGC4, EXO70B1 and BIK1 mainly provide design principles and risk warnings at this stage. The most promising future targets will be genes whose beneficial host functions can be separated from their pathogen-favorable or immune-associated fitness penalties.

## 7. Conclusions and Perspectives

In conclusion, this review highlights a group of immune-associated genes whose disease phenotypes cannot be fully captured by conventional labels such as resistance genes, susceptibility genes, positive regulators or negative regulators. These genes retain recognizable functions in defense or immune homeostasis, yet their disruption can also produce resistance in specific contexts. GSL5 is currently the most informative example, linking pathogen-induced callose deposition, pathogen-dependent immune rewiring and broad-spectrum clubroot resistance in cruciferous plants [10,12]. At present, such dual-state immune regulators remain underexplored. Most examples are still derived from *Arabidopsis* genetics or a limited number of host–pathogen systems, and it is unclear how broadly this phenomenon occurs in crops. Further discovery and mechanistic dissection of similar genes will clarify the conditions under which immune-associated processes become protective, costly or pathogen-favorable. From an applied perspective, GSL5 already shows clear potential for clubroot resistance, whereas other genes discussed here mainly provide conceptual and mechanistic guidance. Continued study of these regulators may expand the conceptual scope of plant immunity and support more precise resistance breeding based on allele design rather than simple gene disruption.

## Data Availability

No new data were created or analyzed in this study. Data sharing is not applicable to this article.

## Abbreviations

The following abbreviations are used in this manuscript:

GSL	Glucan synthase-like
PMR4	Powdery mildew resistant 4
SA	Salicylic acid
JA	Jasmonic acid
NHP	N-hydroxypipecolic acid
ETI	Effector-triggered immunity
PTI	Pattern-triggered immunity
ROS	Reactive oxygen species
DAMP	Damage-associated molecular pattern
PAMP	Pathogen-associated molecular pattern
PRR	Pattern-recognition receptor
RLCK	Receptor-like cytoplasmic kinase
MLO	Mildew resistance locus O
SWEET	Sugars will eventually be exported transporter
MAPK	Mitogen-activated protein kinase
BABA	β-Aminobutyric acid
BIK1	Botrytis-induced kinase 1
CNGC	Cyclic nucleotide-gated channel
DND	Defense, no death
ET	Ethylene
EXO70B1	Exocyst complex component of 70 kDa B1
FLS2	Flagellin-sensitive 2
HR	Hypersensitive response
MAMP	Microbe-associated molecular pattern
MEKK	Mitogen-activated protein kinase kinase kinase
MKK	Mitogen-activated protein kinase kinase
MPK	Mitogen-activated protein kinase
NLR	Nucleotide-binding leucine-rich repeat receptor
PAD4	Phytoalexin deficient 4
TAL	Transcription activator-like
uORF	Upstream open reading frame
